# Autophagy in stem cell aging

**DOI:** 10.1111/acel.12655

**Published:** 2017-08-07

**Authors:** Miren Revuelta, Ander Matheu

**Affiliations:** ^1^ Cellular Oncology Group Biodonostia Institute San Sebastian Spain; ^2^ Ikerbasque, Basque Foundation and CIBERfes Spain

**Keywords:** aging, autophagy, HSC, MSC, stem cell

## Abstract

Aging is responsible for changes in mammalian tissues that result in an imbalance to tissue homeostasis and a decline in the regeneration capacity of organs due to stem cell exhaustion. Autophagy is a constitutive pathway necessary to degrade damaged organelles and protein aggregates. Autophagy is one of the hallmarks of aging, which involves a decline in the number and functionality of stem cells. Recent studies show that stem cells require autophagy to get rid of cellular waste produced during the quiescent stage. In particular, two independent studies in muscle and hematopoietic stem cells demonstrate the relevance of the autophagy impairment for stem cell exhaustion and aging. In this review, we summarize the main results of these works, which helped to elucidate the impact of autophagy in stem cell activity as well as in age‐associated diseases.

## Introduction

Aging is a multifaceted process that leads to alterations in tissue structure and the decline of many functions and activities in mammals. Proteostasis is necessary for most cellular functions, such as genetic replication, catalysis of metabolic reactions, and the immune response. Impairments in proteostasis can lead to toxic aggregations and accumulation of unwanted proteins resulting in cellular dysfunction (Powers *et al*., 2009). The maintenance of tissue homeostasis and the regenerative capacity after an injury depends on tissue‐specific stem cells (Oh *et al*., 2014). The elucidation of the hallmarks of aging by Lopez‐Otin and collaborators identified the impairment of protein homeostasis and stem cell exhaustion as major processes involved in the decline of the regenerative potential capacity linked to the accumulation of age‐associated damage. In particular, loss of proteostasis was considered a primary hallmark, whilst stem cell exhaustion was an integrative hallmark (Lopez‐Otin *et al*., 2013).

Autophagy is a highly conserved catabolic process, essential for this protein quality control, where intracellular components are delivered to lysosomes for self‐degradation (He & Klionsky, 2009). There are three different types of autophagy depending on the signals that induce the pathway, and the mechanism by which the cargo reaches the lysosome: macro‐autophagy (MA), micro‐autophagy, and chaperone‐mediated autophagy (CMA). In particular, MA is involved in recycling long‐lived proteins and cytoplasmic organelles. This process implies the incorporation of proteins, organelles, and cytoplasm in a structure called the autophagosome, which once formed fuses with the lysosome to form autolysosomes and then releasing its content in the lysosomal lumen where it is degraded via acid hydrolases (Zhang & Baehrecke, 2015). Autophagy basal levels are very low under normal conditions, and they are activated in response to stress and extracellular cues. There is an efficient machinery to maintain and induce MA and CMA. There are 30, highly conserved, autophagy‐related genes (*Atg*) that form the core autophagic machinery, and distinct sets of *Atg* genes are involved in the different steps of the autophagy process. Activation of *Atg1/ULK* is indispensable for MA induction as it promotes the formation of a scaffold initiation phagophore necessary for protein recruitment and the initiation of autophagosome formation. Next, the *Atg12* conjugation system, formed by *Atg12*,* Atg5,* and *Atg16*, is necessary for the autophagosome formation, phagophore elongation as well as cargo recognition. The *Atg7* (E1‐like enzyme) is an essential catalyst for autophagosome assembly and cytoplasmic to vacuole transport. The conversion of LC3‐I to LC3‐II is also necessary for autophagosome formation (He & Klionsky, 2009). As a consequence of their role in autophagy machinery, *Atg* genes are involved in many physiological processes such as intracellular quality control, maintenance of cellular and tissue homeostasis, cell differentiation and development, innate and adaptive immunity.

Autophagy is a critical process to maintain cellular homeostasis under normal and stress conditions as its activation promotes cellular survival by maintaining adequate metabolic functions, bioenergetic levels and amino acid pools. It is well known that autophagy can be activated by nutrient starvation but also other triggers such as exercise, infections, or oxidative stress (Galluzzi *et al*., 2014). Genetic studies of *Atg5* ^−/−^ and *Atg7* ^−/−^ mice revealed that the lack of autophagy is lethal within the first postnatal hours as autophagy is required to overcome the perinatal starvation period (Kuma *et al*., 2004; Komatsu *et al*., 2005). Thus, autophagy is very sensitive to nutrient deprivation and in this condition, autophagosome formation is regulated by two main signaling cascades, mTOR and Ras‐cAMP‐PKA pathways, and their inhibition stimulates autophagy in the presence of nutrients (Rodgers *et al*., 2014).

## Impact of autophagy in longevity and aging

Aging results from the accumulation of cellular damage promoted by chronic stresses of small magnitude. Therefore, being a sensor of stress, autophagy has been linked to aging. Several studies have described a decline in autophagy activity (measured by altered autophagic flux, LC3 and p62 protein expression) as well as expression of autophagy gene such as *Atg1, Atg5, Atg6, Atg7, Atg8, and Atg12* in response to aging in several animal models and human tissues. The majority of these works have focused on MA, the most studied form of autophagic process*,* but there have also been studies showing a decline in CMA, particularly in the liver and the central nervous system, which have been linked to decreased function in these organs (Cuervo, 2008; Rubinsztein *et al*., 2011). Longevity studies with gain and loss of autophagy genes in animal models such as yeast, *C. elegans, Drosophila* and mice, support a direct role for autophagy in the aging process. For example, genetic experiments demonstrate a requirement for key autophagy regulators such as Atg1/*ULK1*,* Atg7*,* Atg8*,* Atg11,* or *Beclin1* as their inactivation or reduction in levels accelerated aging and reduced lifespan in different animal models. These phenotypes have been associated with appearance of age‐associated pathologies such as damaged mitochondria, reduced cardiac performance, lipid accumulation, and muscle degeneration. In contrast, overexpression of *Atg5* or *Atg12*, among others, extended life span in several species and delayed the decline of multiple features of aging such as mitochondrial maintenance or motor function (Madeo *et al*., 2010, 2015). Clear proof for the impact of CMA in aging came with the generation of transgenic mice harboring an extra copy of LAMP2a, a CMA receptor in lysosomal membrane, that specifically targeted to the liver, and that demonstrated an improved cellular homeostasis and hepatic function in aged mice (Zhang & Cuervo, 2008). In addition, genetic studies have revealed that impairment of MA, via inactivation of various *Atg* genes, is involved, along with other causative factors, in age‐associated pathologies, including neurodegenerative diseases, cancer, cardiac malfunction, reduced muscle mass, and sarcopenia (Hara *et al*., 2006; Rubinsztein *et al*., 2011; Zhou *et al*., 2017). A decline in CMA activity has been associated with age‐related pathologies such as Alzheimer's and Parkinson's diseases (Cuervo & Wong, 2014). Further support for a link between autophagy and aging comes from studies involving caloric restriction, the most effective strategy to induce autophagy, that is also the most efficient and reproducible intervention known to increase lifespan and delay aging in several organisms ranging from yeast to primates (Lopez‐Otin *et al*., 2013). Caloric restriction induces the inhibition of mTOR complex 1 and activation of AMPK, which in turn results in the activation of the ULK1 complex. Caloric restriction also stimulates SIRT1, which is known to activate many essential autophagic proteins. Interestingly, pharmacological stimulation of MA, via the administration of the mTOR inhibitor rapamycin (an agent that mimic the effects of caloric restriction), extends lifespan and presents anti‐aging effects in several animal models such as yeast, flies and mice (Madeo *et al*., 2010; Lopez‐Otin *et al*., 2013). Similarly, spermidine, a natural inducor of autophagy, maintains long‐lasting immunity and slows down progression of neurodegenerative diseases associated with age. These autophagy‐dependent effects are dependent on *Atg7* gene function (Puleston *et al*., 2014; Bhukel *et al*., 2017). All this evidence supports the assertion that autophagy is involved in longevity, aging and development of age‐associated pathologies and has encouraged the scientific community to identify the precise role and molecular mechanisms of autophagy in aging, as targeting autophagy could be a novel therapy against aging and age‐related diseases.

## Impact of autophagy in stem cell activity and aging

**Figure 1:**
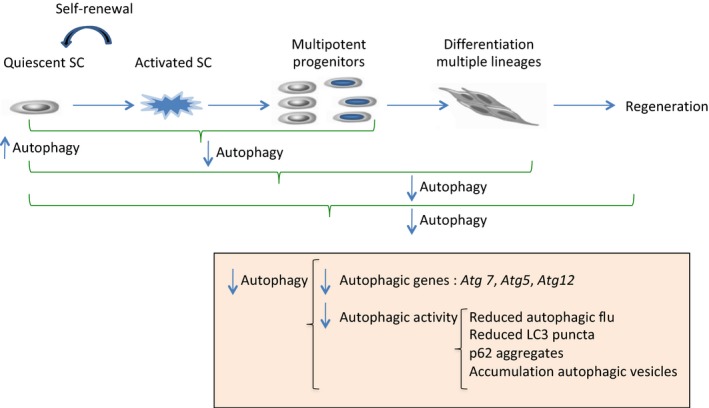
Autophagy in stem cell function. Under normal conditions, basal autophagy is high in quiescent stem cells (SCs), at least in MSCs and HSCs, and MA is necessary for the maintenance of quiescence state and self‐renewal activity. Under stress conditions such as aging, there is a decline in stem cell numbers and activity that impairs stem cell activation, lineage differentiation and consequently, there is a failure in regenerative capacity. These processes correlates with reduced MA as the levels of several autophagy machinery genes are decreased in aged SCs. Autophagic function, measured by different methods, is also impaired in aged SCs. Maintenance of self‐renewal, SC activation, differentiation ability, and regeneration capacity in muscle and hematopoietic system is mediated by MA genes such as *Atg5*,* Atg7,* or *Atg12* (Garcia‐Prat *et al*., 2016; Ho *et al*., 2017).

How autophagy decreases with age remains unclear and under intense investigation. Recent works carried on aged muscle stem cells (MSC) and hematopoietic stem cells (HSC) have revealed the impairment of MA in stem cell activity with aging (Fig. 1A,B). Moreover, these studies have confirmed that correct functioning of MA is necessary to maintain the appropriate blood system and muscle development and to allow adult stem cell to survive under metabolic stress (Garcia‐Prat *et al*., 2016; Ho *et al*., 2017).

**Figure 2:**
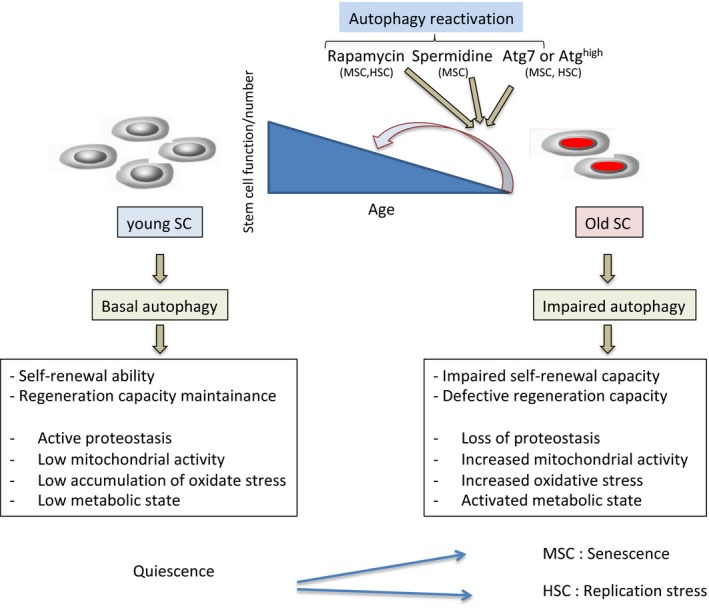
Effect of aging in young and old stem cells in terms of autophagy activity. There is altered autophagy activity between young and old SCs, at least in MSCs and HSCs. Aged SCs present decreased autophagy activity (specially reduced MA) that correlates and promotes impaired self‐renewal, SC exhaustion and defective regeneration capacity. Mechanistically, decreased autophagy activity induces loss of proteostasis, increased mitochondrial activity, increased oxidative stress, and activated metabolic state in both MSCs and HSCs. However, there are also differences between MSCs and HSCs in terms of autophagy function with aging. Autophagy impairment causes MSC to undergo senescence whilst it alters HSCs metabolism and replication stress response. Pharmacological reactivation (with rapamycin and spermidine) or re‐establishment of MA (with genetic overexpression of *Atg7*) reverses SC function and restores regenerative capacity. Similarly, a proportion of high autophagy SCs maintains self‐renewal and preserves regenerative potential (Garcia‐Prat *et al*., 2016; Ho *et al*., 2017).

These studies suggest that MSC and HSC lose their regenerative abilities when they reach an advanced age and that autophagy is deficient in the old stem cell population (Fig. 2). This deficiency results in the accumulation of MA vesicles, increased intracellular p62 protein levels, increased LC3II expression and ubiquitin‐positive inclusions. Moreover, when treating these cells with rapamycin and spermidine, MA capacity is restored, ameliorating autophagy‐mediated deficiencies in both systems (Garcia‐Prat *et al*., 2016; Ho *et al*., 2017). These works firmly establish the link between basal autophagy and stem cell aging.

In a previous study from the Passegué team, they found an increase in basal autophagy with aging in HSC and enhanced maintenance of the regenerative capacity (Warr *et al*., 2013). In a recent work, the same group found that approximately 30% of aged HSCs exhibited high autophagy levels, maintaining a low metabolic state and strong long‐term regeneration potential similar to young HSCs. However, the remaining population of aged HSCs showed loss of autophagy, which causes activated metabolic state, accelerated myeloid differentiation, and impaired HSCs self‐renewal activity and regenerative potential (Ho *et al*., 2017). To reach these conclusions, the authors directly inactivated MA in the blood system taking advantage of conditional *Atg12* ^*Flox/Flox*^ and *Atg5* ^*Flox/Flox*^ mouse models. Indeed, *Atg* mutant mice presented features of premature blood aging in adult stage such as increased cellularity in the peripheral blood and spleen, skewed ratio of circulating myeloid versus lymphoid cells, phenotype which resembled the myeloid‐bias characteristic in old mice. On the contrary, *Atg* mutant mice maintained similar number of phenotypic HSCs (Lin−/c‐Kit+/Sca‐1+/Flk2−/CD48−/CD150+) than *wild‐type* mice over time (Ho *et al*., 2017). Moreover, transplantation assays with purified adult autophagy‐deficient HSCs showed decreased number of regenerated HSCs, impaired engraftment and myeloid‐biased lineage distribution among other features in primary transplantation, and presented decreased self‐renewal activity upon secondary transplantation, which was similar to the activity from old animals (Ho *et al*., 2017). Transplantation of autophagy‐deficient bone marrow cells from adult and aged mice resulted in significantly exacerbated age‐related decline in donor chimerism and myeloid‐biased lineage distribution (Ho *et al*., 2017). These results demonstrate the intrinsic cellular role of autophagy as being critical for HSC function during aging and under conditions of intense regenerative stress.

A study from the Muñoz‐Cánoves laboratory identified autophagy as the most prevalent pathway in muscle quiescent satellite cells, revealed a marked decline of the expression of autophagic genes in this population with aging, and demonstrated that the loss of autophagy results in a decline in both the number and function of satellite cells as well as muscle activity, which can be restored by autophagy re‐establishment (Garcia‐Prat *et al*., 2016). The authors taking advantage of GFP‐LC3 transgenic mice proved that young quiescent MSCs have a constitutive autophagy activity that diminishes with aging and that the impairment that occurs in old cells is related to their lack in autophagosome formation capacity. Similar results were obtained in aged human cells. Moreover, the authors using constitutive and conditional *Atg7* ^*Flox/Flox*^ directly targeted to MSCs showed that genetic impairment of macro‐autophagy in young MSCs promotes the entry into premature senescence by loss of proteostasis, increased mitochondrial dysfunction and oxidative stress. MA deficient young MSCs also showed reduced activation and expansion capacity as well as decreased size of regenerating fibers in response to muscle injury (Garcia‐Prat *et al*., 2016). They restored MSC regenerative capacity re‐establishing MA, together firmly confirming the cell‐intrinsic role of autophagy in MSC function with aging. Previously, another study revealed that MA, in particular *Atg7*, is necessary to supply the bioenergetics demands that the activation of quiescent MSCs requires after injury, an action mediated by SIRT1 (Tang & Rando, 2014).

These studies confirm that autophagy maintains stemness in MSC and HSCs; however, the maintenance of this feature seems to be different in each niche. Garcia‐Prat *et al*. demonstrated that loss of autophagy induces premature aging causing stem cells to undergo senescence as a consequence of altered mitophagy and increased reactive oxidant species (ROS) production. Indeed, they analyzed the effect of ROS *in vivo*, treating old GFP‐LC3 mice with an intraperitoneal injection of the antioxidant Trolox resulting in increased autophagy and decreased expression of senescence markers such as p16^INK4a^ and senescence associated‐β‐galactosidase + cells. Moreover, *in vitro* treatment with Trolox or *p16* ^*INK4a*^ knockdown in MSCs derived from aged mice and autophagy mutant mice restored proliferation, reduced senescence entry and increased their regenerative potential (Garcia‐Prat *et al*., 2016). On the other hand, Ho *et al*. showed that autophagy regulates stemness via stem cell metabolism, replication stress and mitochondrial activity. Indeed, *Atg12* mutant young HSCs presented more active mitochondria, increased oxidative phosphorylation (OXPHOS) with decreased glycolysis and several changes in cellular features, all of them phenotypes associated with aged HSCs. As a consequence of being more metabolically active, autophagy‐deficient HSCs showed increased levels of ROS, elevated protein synthesis rates, and increased cell cycle activity resulting in the loss of quiescence and acceleration of myeloid differentiation, which occurs in old HSCs. Interestingly, autophagy inhibitor bafilomycin A, but not the treatment with the antioxidant N‐acetylcysteine, rescued the myeloid differentiation and expansion deficits. Finally, the authors showed that autophagy mutant HSCs present a significantly altered DNA methylation profile, evidence consistent with the idea that loss of autophagy‐mediated changes in fate decisions is the consequence of an epigenetic reprogramming that alters gene expression in HSCs, with amplification of transcriptional changes in downstream progenitors (Ho *et al*., 2017).

## Discussion

In the last decade, there have been incredible advances in understanding stem cell aging and the molecular mechanisms underlying this process. Indeed, the induction of p16^Ink4a^ senescence marker (Janzen *et al*., 2006; Krishnamurthy *et al*., 2006; Molofsky *et al*., 2006), the activation of p38 MAPKinase (Bernet *et al*., 2014; Cosgrove *et al*., 2014), and STAT3 (Price *et al*., 2014; Tierney *et al*., 2014) pathways are nowadays well‐recognized critical regulators of stem cell activity and there are different strategies currently being tested targeting them as therapy against stem cells and age‐related diseases (Oh *et al*., 2014). The novel works of Garcia‐Prat and Ho offers relevant new information regarding the autophagy malfunctioning in aged stem cell and the effect of reversing this disorder in two independent stem cell niches. Whether these results can be translated to other stem cell niches will be surely determined in the future. In addition, it will also be important to elucidate whether CMA or micro‐autophagy play any role in stem cell aging. These works uncovered the requirement of correct autophagy activity for stem cell function and postulated the pharmacological restoration of autophagy as a novel strategy to boost stem cell activity for regenerative medicine and aging. The discovery that rapamycin extends mammalian lifespan and activates autophagy is promising because it represents the first demonstration of pharmacological extension of maximal lifespan linked to autophagy in mammalian species. Rapamycin and its derivatives are FDA‐approved, are used clinically to prevent organ rejection after kidney transplantation, and are also being tested in clinical trials for the treatment of several cancers and neurodegenerative disorders. Therefore, considerable experience exists with the application of this compound in the clinic. However, it is important to first unravel whether the rapamycin‐promoted lifespan extension is dependent on autophagy effects or not.

## Funding

This work is supported by grants from the Instituto Salud Carlos III and FEDER Funds (CP16/00039, PI16/01580, DTS16/0184), Diputacion Foral Gipuzkoa and Marie Curie CIG (2012/712404) to the laboratory of AM.

## Conflict of interest

The authors declare no competing financial interests.
